# PPAR-gamma activation is associated with reduced liver ischemia-reperfusion injury and altered tissue-resident macrophages polarization in a mouse model

**DOI:** 10.1371/journal.pone.0195212

**Published:** 2018-04-04

**Authors:** Ivan Linares, Kaveh Farrokhi, Juan Echeverri, Johan Moritz Kaths, Dagmar Kollmann, Matyas Hamar, Peter Urbanellis, Sujani Ganesh, Oyedele A. Adeyi, Paul Yip, Markus Selzner, Nazia Selzner

**Affiliations:** 1 Multi Organ Transplant Program, Toronto General Hospital, Toronto, ON, Canada; 2 Consejo Nacional de Ciencia y Tecnología, México City, México; 3 Institute of Medical Science, University of Toronto, Toronto, ON, Canada; 4 Department of Immunology, University of Toronto, Toronto, ON, Canada; 5 Department of Pathology, Toronto General Hospital, Toronto, ON, Canada; 6 Laboratory of Medicine and Pathobiology, Toronto General Hospital, Toronto, ON, Canada; IDIBAPS Biomedical Research Institute, SPAIN

## Abstract

**Background:**

PPAR-gamma (γ) is highly expressed in macrophages and its activation affects their polarization. The effect of PPAR-γ activation on Kupffer cells (KCs) and liver ischemia-reperfusion injury (IRI) has not yet been evaluated. We investigated the effect of PPAR-γ activation on KC-polarization and IRI.

**Materials and methods:**

Seventy percent (70%) liver ischemia was induced for 60mins. PPAR-γ-agonist or vehicle was administrated before reperfusion. PPAR-γ-antagonist was used to block PPAR-γ activation. Liver injury, necrosis, and apoptosis were assessed post-reperfusion. Flow-cytometry determined KC-phenotypes (pro-inflammatory Nitric Oxide +, anti-inflammatory CD206+ and anti-inflammatory IL-10+).

**Results:**

Liver injury assessed by serum AST was significantly decreased in PPAR-γ-agonist versus control group at all time points post reperfusion (1hr: 3092±105 vs 4469±551; p = 0.042; 6hr: 7041±1160 vs 12193±1143; p = 0.015; 12hr: 5746±328 vs 8608±1259; p = 0.049). Furthermore, liver apoptosis measured by TUNEL-staining was significantly reduced in PPAR-γ-agonist versus control group post reperfusion (1hr:2.46±0.49 vs 6.90±0.85%;p = 0.001; 6hr:26.40±2.93 vs 50.13±8.29%; p = 0.048). H&E staining demonstrated less necrosis in PPAR-γ-agonist versus control group (24hr:26.66±4.78 vs 45.62±4.57%; p = 0.032). The percentage of pro-inflammatory NO+ KCs was significantly lower at all post reperfusion time points in the PPAR-γ-agonist versus control group (1hr:28.49±4.99 vs 53.54±9.15%; p = 0.040; 6hr:5.51±0.54 vs 31.12±9.58%; p = 0.009; 24hr:4.15±1.50 vs 17.10±4.77%; p = 0.043). In contrast, percentage of anti-inflammatory CD206+ KCs was significantly higher in PPAR-γ-agonist versus control group prior to IRI (8.62±0.96 vs 4.88 ±0.50%; p = 0.04). Administration of PPAR-γ-antagonist reversed the beneficial effects on AST, apoptosis, and pro-inflammatory NO+ KCs.

**Conclusion:**

PPAR-γ activation reduces IRI and decreases the pro-inflammatory NO+ Kupffer cells. PPAR-γ activation can become an important tool to improve outcomes in liver surgery through decreasing the pro-inflammatory phenotype of KCs and IRI.

## Introduction

Ischemia and reperfusion injury (IRI) is an important problem during solid organ transplantation, trauma, hypovolemic shock, and elective liver resection when inflow occlusion or total vascular exclusion is used to minimize blood loss. The liver IRI induces hepatocyte injury and activation of an inflammatory signaling cascade resulting in graft dysfunction and increased morbidity and mortality after surgery [1]. Histopathologic changes include cellular swelling, vacuolization, endothelial cell disruption, neutrophil infiltration, hepatocellular necrosis and apoptosis [2–4]. IRI is a dynamic process, in which the innate and adaptive immune inflammatory responses play an essential role in developing early allograft dysfunction or primary non-function[5, 6]. Kupffer cells (KCs), the resident macrophages of the liver are an important part of the innate immune response and also represent the largest fixed population of macrophages in the body, comprising 40–65% of liver non-parenchymal cells. The activation of KCs is thought to initiate hepatic IRI, and it is followed by the release of a series of pro-inflammatory cytokines such as tumor necrosis factor α (TNF-α) and interleukin (IL-1β), the expression of cell adhesion factors, the production of reactive oxygen species, prostanoids and nitric oxide (NO) [7, 8]. TNF-α is a major effector on hepatocyte and endothelial injury inducing leukocyte chemotaxis, activating neutrophils, and generating free radicals, as well as inducing mitochondrial toxicity and apoptosis. In addition, the excessive macrophage NO production by iNOS contributes to the hepatic oxidative damage in IRI and has been related to the pro-inflammatory macrophage population [8, 9], [10]. In contrast, the KC population that expresses the surface marker CD206 and synthesizes IL10 has been associated with a decrease in inflammatory response and classified as part of the anti-inflammatory macrophage population [11]. Moreover, due to the array of distinct types of liver cells in close proximity to each other allowing for cell-cell interactions, the KCs are intimately involved in liver response to stress and endogenous ligands released from injured or necrotic hepatocytes which are recognized by KCs through surface receptors, thus initiating the signaling cascade resulting in inflammation and organ damage at the early phase of the IRI event [12], [13], [14].

The peroxisome proliferator-activated receptor-γ (PPAR-γ) is a member of the nuclear receptor family of transcription factors, a large and diverse group of proteins that mediate ligand-dependent transcriptional activation and repression [15]. This receptor is highly expressed in macrophages and its activation has been linked to the up-regulation of the anti-inflammatory macrophage phenotype and down-regulation of the pro-inflammatory macrophage phenotype that as a consequence leads to a decrease of the inflammatory response [16], [17], [18]. It is important to understand macrophage polarization as a spectrum in which there are not pure M1 or M2 macrophage populations and that these phenotypes include transitions according to the signal that they receive. Resident macrophages are an essential component in the liver. KCs represent the liver-resident population of macrophages and can be distinguished from the monocyte-derived macrophages through different surface markers. These populations are also different in origin being the KCs derived from the yolk sac and the monocytes from bone marrow. KCs have been determined to be predominantly of embryonic origin and maintained through self-renewal in the steady state with some contribution from bone marrow monocytes [19], [20]. PPAR-γ agonists have shown a beneficial effect on IRI in cerebral, cardiac and renal tissue [21], [22], [23], [24], [25], [26] suggesting macrophage polarization as the potential mechanism of action in some of these models [27]. Prior studies have demonstrated a protective effect of PPAR-γ agonists on hepatocyte injury. However, the role of PPAR-γ activation on KC polarization in conjunction with liver ischemia-reperfusion injury has not been assessed yet.[28], [29]. In this study, we determined the effects of PPAR-γ on KC polarization in liver tissue and its impact on hepatic ischemia and reperfusion injury.

## Material and methods

### Reagents

Rosiglitazone (PPAR-γ agonist) and GW9662 (PPAR-γ antagonist) were purchased from Sigma-Aldrich laboratory (St Louis, MO, USA) and Cayman Chemical laboratory (Ann Arbor, MI, USA) respectively.

### Animals

Male wild-type C57BL/6 (10–12 weeks old) were purchased from Ontario Cancer Institute. All animals were maintained in a laminar-flow specific pathogen-free atmosphere at the Princess Margaret Cancer Research Tower. The Animal Care Committee of the Toronto General Hospital approved the experiments. Animals received human care in observance with the “Principles of Laboratory Animal Care” formulated by the National Society for Medical Research, the “Guide for the Care of Laboratory Animals” published by the National Institute of Health, and the “Animal Research-Reporting of In Vivo Experiments” (ARRIVE) guidelines.

### Vert-X mouse

Mice were purchased from Jackson laboratory and breed in Ontario Cancer Institute to extend the colony. To create this mouse a targeting vector is designed to place an internal ribosome entry site (IRES)-enhanced green fluorescent protein (*eGFP*) fusion protein, downstream of exon 5 of the interleukin 10 (*Il10*) gene [30].

### Experimental design

A nonlethal model of segmental (70%) hepatic warm ischemia was used. Under isoflurane (inhalation) anesthesia, a midline laparotomy was performed. Liver hilum was dissected free of surrounding tissue and structures in the portal triad (hepatic artery, portal vein, and bile duct) to the left and median liver lobes were occluded with a microvascular clamp for 60 min, and reperfusion was initiated by removal of the clamp. Throughout the ischemic interval, evidence of ischemia was confirmed by visualizing the pale blanching of the ischemic lobes. The clamp was then removed and gross evidence of reperfusion based on immediate color change was assured before closing the abdomen with a continuous 4–0 diameter polypropylene suture. Five animals (n = 5) per group were sacrificed at predetermined time points (1hr, 6hr, 12hr and 24hr) after reperfusion to obtain serum and liver samples. These time-points were chosen to evaluate and show the kinetic curve of AST. However, prior publications on rodent models of liver ischemia-reperfusion injury have demonstrated that the peak of AST after reperfusion is better observed at 6hrs following reperfusion with concentration reaching almost normal levels by 24-48hrs following reperfusion. The efficacy of therapeutic interventions is best detected by reducing the injury peak at 6hrs [31], [32] [33], [34], [35], [36], [37]. Additionally, to represent baseline (BL) time point, five animals (n = 5) underwent anesthesia, laparotomy, and exposure of the portal triad without hepatic ischemia and then sacrificed to collect serum and liver samples.

Either the absence of ischemic color changes or the lack of response to reperfusion was a criterion for immediate sacrifice and exclusion from further analysis.

Rosiglitazone (PPAR-γ agonist) was dissolved in DMSO/ 0.9%Saline (Ratio 1:6) and administrated at 3mg/kg. Rosiglitazone (RGZ) or vehicle (control) in the first set of experiments was administrated 24 and 1hr before reperfusion by intraperitoneal injection. In an additional group for the same set of experiments GW-9662 (PPAR-γ antagonist) was intraperitoneally injected 30 minutes prior to Rosiglitazone to assess inhibition of PPAR-γ activation. Additionally, the second set of experiments was utilized to evaluate the results of single intraperitoneal RGZ (3mg/kg) or vehicle (control) injection only after reperfusion. For this second set of experiments the inhibition of PPAR-γ activation was also evaluated with the prior injection of GW-9662 30 minutes before RGZ administration.

### Liver injury

Serum aspartate aminotransferase (AST) was measured 1hr, 6hr, 12hr and 24hr after reperfusion to evaluate hepatic injury after reperfusion. The ARCHITECT c8000 clinical chemistry analyzer (Abbott Diagnostics, Abbott Park, IL, USA) was used to determine AST levels in mouse serum.

### Histopathology

The whole left lateral lobe was taken and was formalin-fixed. Samples were embedded in paraffin and cut into 6-um thick sections. Tissues were stained with H&E, and slides were scanned and assessed for necrosis by using Aperio image scope software [38], [39]

### Immunohistochemistry staining

Liver samples were embedded in paraffin and cut into 6-um thick sections. Staining for F4/80 was performed and assessed as a surface marker of macrophages (MCA497, Bio-RAD laboratories, Hercules, California, USA) [40]. Terminal deoxynucleotidyl transferase dUTP nick end labeling (TUNEL)(Bio-11-dUTP, Cedarlane Laboratories, Burlington, NC, USA; dATP, dCTP, dGTP and DNA Polymerase, Promega laboratories, Madison, WI, USA) [41] and cleaved caspase 3 immunohistochemistry assay (ASP175 antibody, Cell Signaling Technology) [42] was used to assess degree of apoptosis. Slides were scanned and evaluated by using Aperio ImageScope software [39].

### Objective image analysis

High-resolution whole slide scanning for histopathology specimens was performed with the ScanScope AT2 (Leica Biosystems Inc, Buffalo Grove, IL, USA). Aperio ImageScope (Leica Biosystems Inc, Buffalo Grove, IL, USA) software was used to objectively quantify positive staining in the case of immunohistochemistry and to delineate necrotic areas in the necrosis assessment [39].

### Flow-cytometry

Reagents and antibodies used for flow cytometry were CD45-BV650 clone 30-F11, F4/80-PECy7 clone BM8, CD11b-PE clone M1/70, CD206-FITC C068C2, FITC Rat IgG2a, κ Isotype Ctrl—Clone RTK2758 (Biolegend, San Diego, CA, USA), DAF-FM diacetate (ThermoFisher Scientific, Burlington, ON, Canada), Fixable Viability Dye eFluor® 450 (eBioscience/ThermoFisher Scientific, Burlington, ON, Canada).

Livers were digested and live cells were gated on. The pro-inflammatory NO+ KCs population was evaluated by the following combination: CD45+, CD11b^lo^, F4/80^hi^, DAF-FM + (the marker for nitric oxide production). The anti-inflammatory CD206+ KCs population was assessed by the following markers: CD45+, CD11b^lo^, F4/80^hi^, CD206 + (mannose receptor). Additionally, the anti-inflammatory IL-10+ KCs population was assessed by flow cytometry quantifying GFP expression in KCs of Vert-X mouse (CD45+, CD11b^lo^, F4/80^hi^, GFP-IL10+). The pro-inflammatory-NO+/ anti-inflammatory-CD206+ Kupffer cells ratio was calculated by dividing the percentage of cells positive for NO by the total of cells positive for CD206.

### Cytokine measurement

Serum concentrations of TNF-alpha, IL-6, and IL-10 were determined by using LEGENDplex^TM^ multi-analyte bioassay kit according to manufacturer´s protocol (Biolegend®, San Diego, CA, USA)

### Statistical analysis

Results are reported as mean and standard error of the mean (SEM). The data were analyzed with the SPSS 20 statistic package. Mann-Whitney U test was used to analyze continues variables. The results were considered significant at the level of p <0.05.

## Results

### PPAR-γ activation reduces hepatic IRI

**Fig 1:**
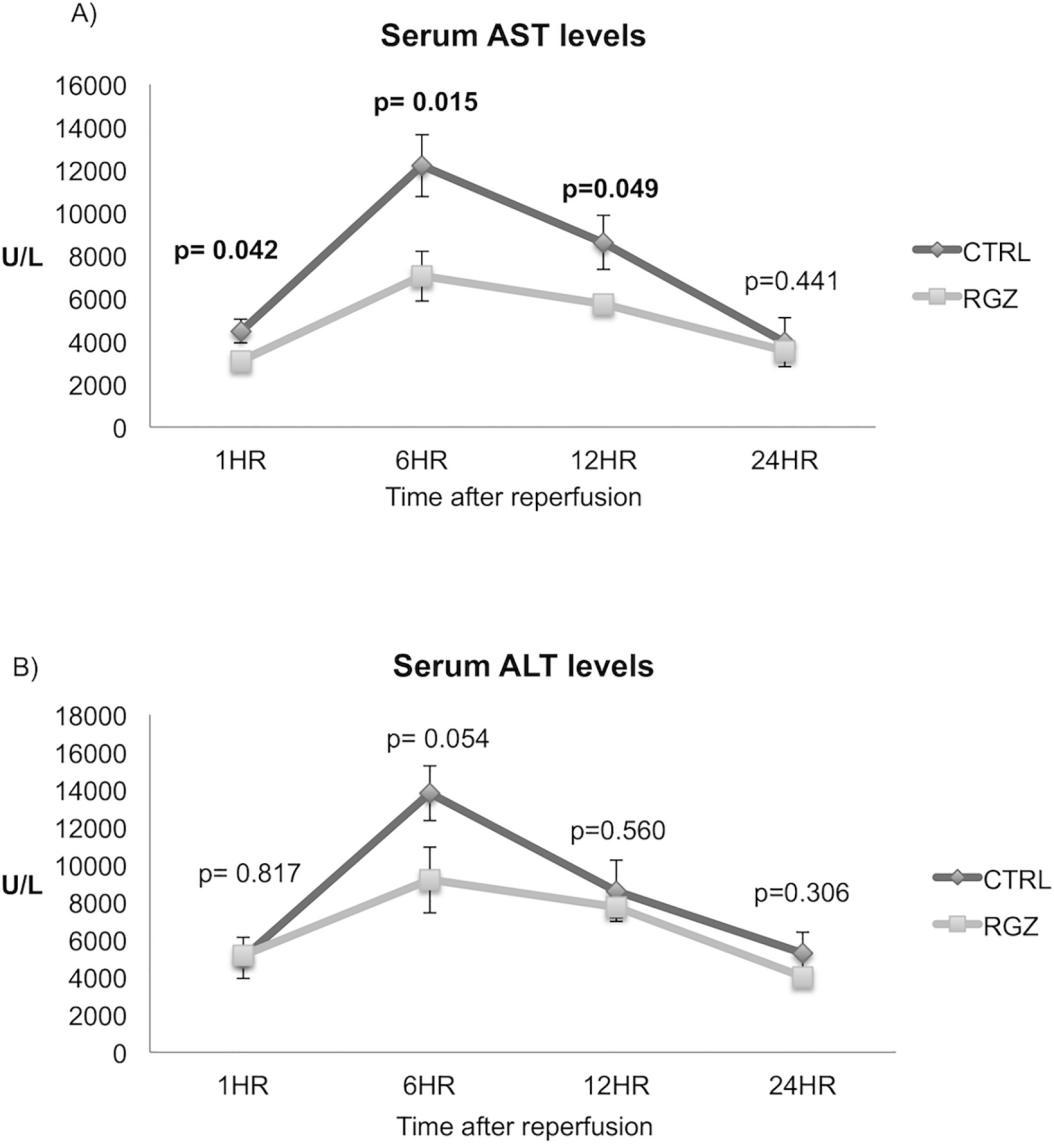
Hepatocellular injury after 60min of ischemia and 1, 6, 12 and 24hrs of reperfusion. Significant lower AST levels were found in the RGZ vs CTRL group at 1hr (3092±105 vs 4469±551; p = 0.042), 6hr (7041±1169 vs 12192±1443; p = 0.015) and 12hr (5746±328 vs 8609±1259; p = 0.049) after reperfusion (A). RGZ vs control group showed a trend to a significant difference at 6hrs post-reperfusion in ALT (9185±1754 vs 13823±1465; p = 0.054) (B). Abbreviations: AST-aspartate aminotransferase, ALT-alanine aminotransferase, RGZ-Rosiglitazone, CTRL-Control. Five experiments (n = 5) per group per time point were performed. Results are shown as mean ± SEM, Mann-Whitney U test.

The severity of hepatic reperfusion injury was assessed by measurement of serum AST. AST level was significantly lower in the Rosiglitazone (RGZ) vs control group as early as 1hr post-perfusion (3092±105 vs 4469±551 U/L; p = 0.042), with a peak at 6hr post-reperfusion (7041±1160 vs 12193±1443 U/L; p = 0.015). This difference remained significant up to 12hr post-reperfusion (5746±328 vs 8608±1259 U/L, p = 0.049)(Fig 1). RGZ vs control group demonstrated a trend to a significant difference at 6hrs post-reperfusion for ALT levels (9185±1754 vs 13823±1465; p = 0.054) as shown in Fig 1.

### PPAR-γ activation reduces hepatic apoptosis and necrosis following IRI

**Fig 2:**
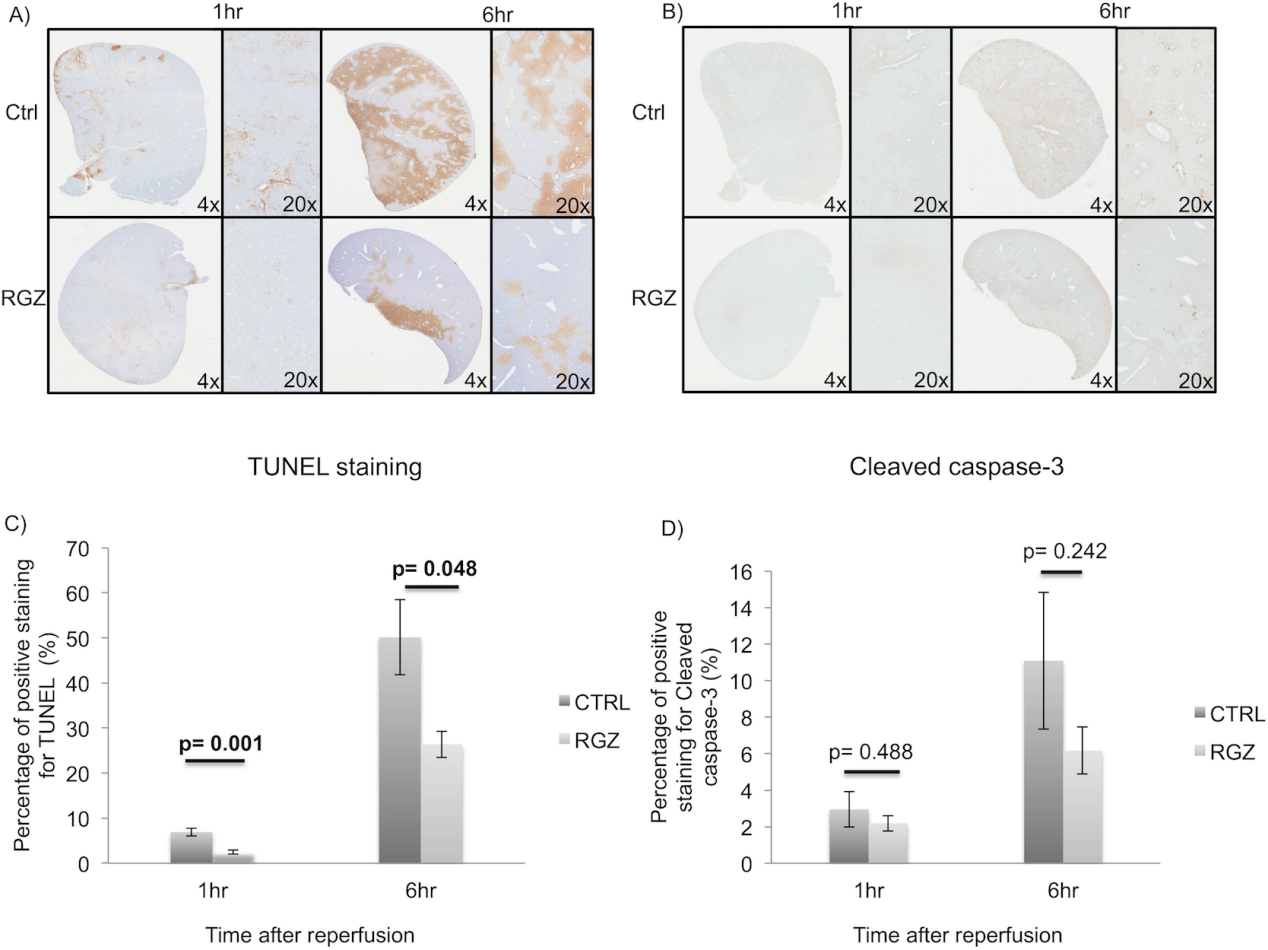
Level of apoptosis. Representative images of TUNEL(A) and Cleaved Caspase-3(B) immunohistochemistry showing a lower grade of apoptosis in RGZ vs Control group at 1hr and 6hr post-reperfusion. Significant lower TUNEL-staining was found in the RGZ vs CTRL group at 1hr (2.46±0.49 vs 6.90±0.85%; p = 0.001) and 6hrs (26.40±2.93 vs 50.13±8.29%; p = 0.048) post-reperfusion by image analysis(C). Cleaved caspase-3 immunohistochemistry also demonstrated less staining in the RGZ group vs control group, the difference was assessed by image software analysis (D). Abbreviations: TUNEL-Terminal deoxynucleotidyl transferase(TdT) dUTP Nick-End-Labeling, RGZ-Rosiglitazone, Ctrl-Control. Five experiments (n = 5) per group per time point were performed. Results are shown as mean ± SEM, Mann-Whitney-U-test.

TUNEL staining was significantly reduced in the RGZ treated group when compared to control at 1hr and 6hr after reperfusion (1hr: 2.46±0.49 vs 6.90±0.85%; p = 0.001; 6hrs: 26.40±2.93 vs 50.13±8.29%; p = 0.048). Cleaved caspase-3 staining was also found to be lower in the RGZ treated group versus control after reperfusion without reaching significant difference (1hr: 2.18±0.42 vs 2.95±0.97%; p = 0.488; 6hrs: 6.17±1.28 vs 11.09±3.74%, respectively, p = 0.242) (Fig 2).

**Fig 3:**
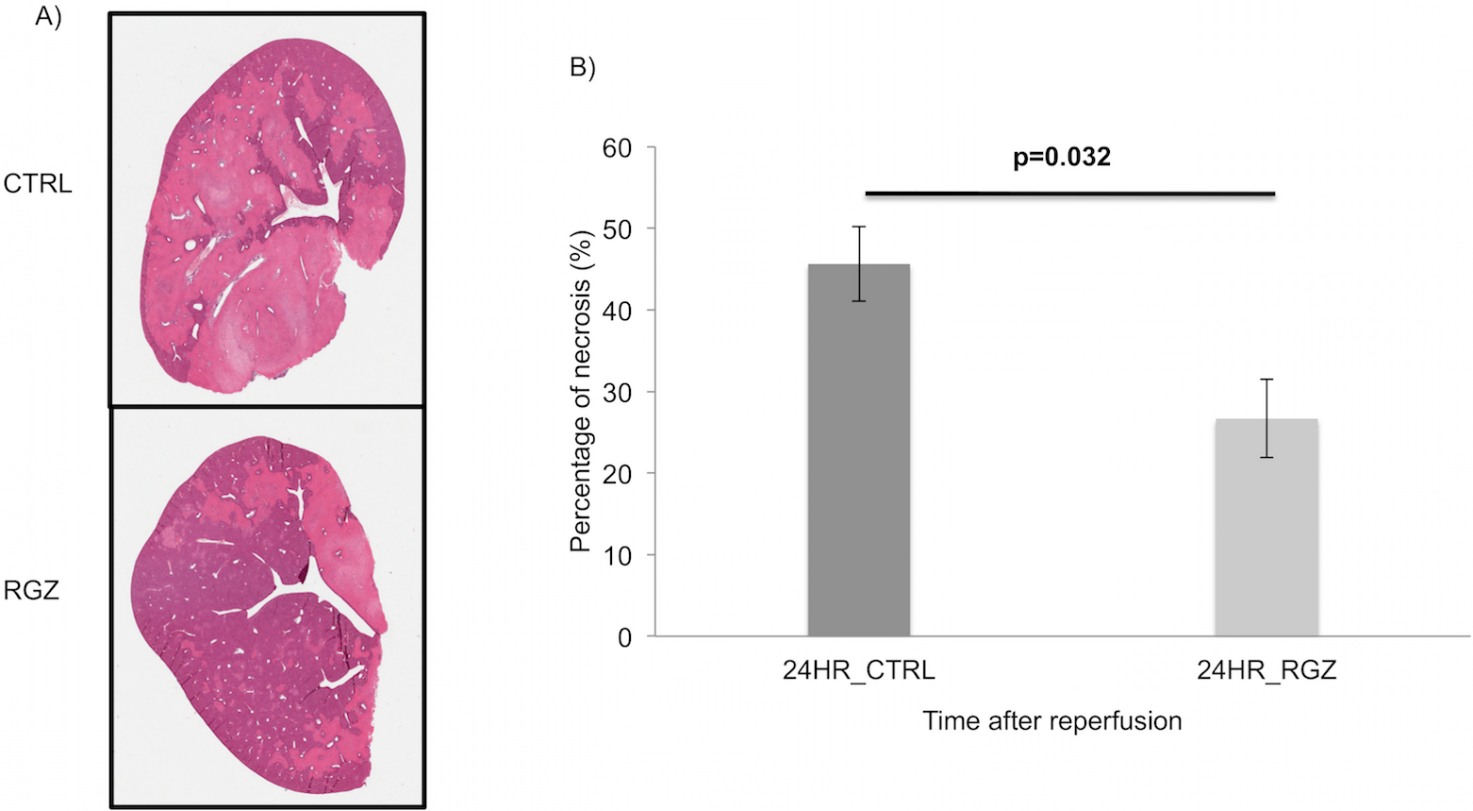
Hepatic necrosis. Representative pictures of the left lateral lobe with H&E staining showing a less necrotic area in the RGZ vs control group (A) 24hrs after reperfusion (26.66±4.78 vs 45.62±4.57%; p = 0.032). Image software analysis demonstrated that this difference was significant (B). Abbreviations: CTRL Control, RGZ Rosiglitazone. Five experiments (n = 5) per group per time point were performed. Results are shown as mean ± SEM, Mann-Whitney U test.

We next investigated the severity of hepatic necrosis 24hr after reperfusion. PPAR-γ activation led to significantly lower percentage of necrotic tissue when compared to the non-treated group (26.66±4.78 vs 45.62±4.57%; p = 0.032) (Fig 3).

Overall these data demonstrated less degree of apoptosis and necrosis in the RGZ treated group.

### Effect of PPAR-γ activation on TNF-α and IL-10

**Fig 4:**
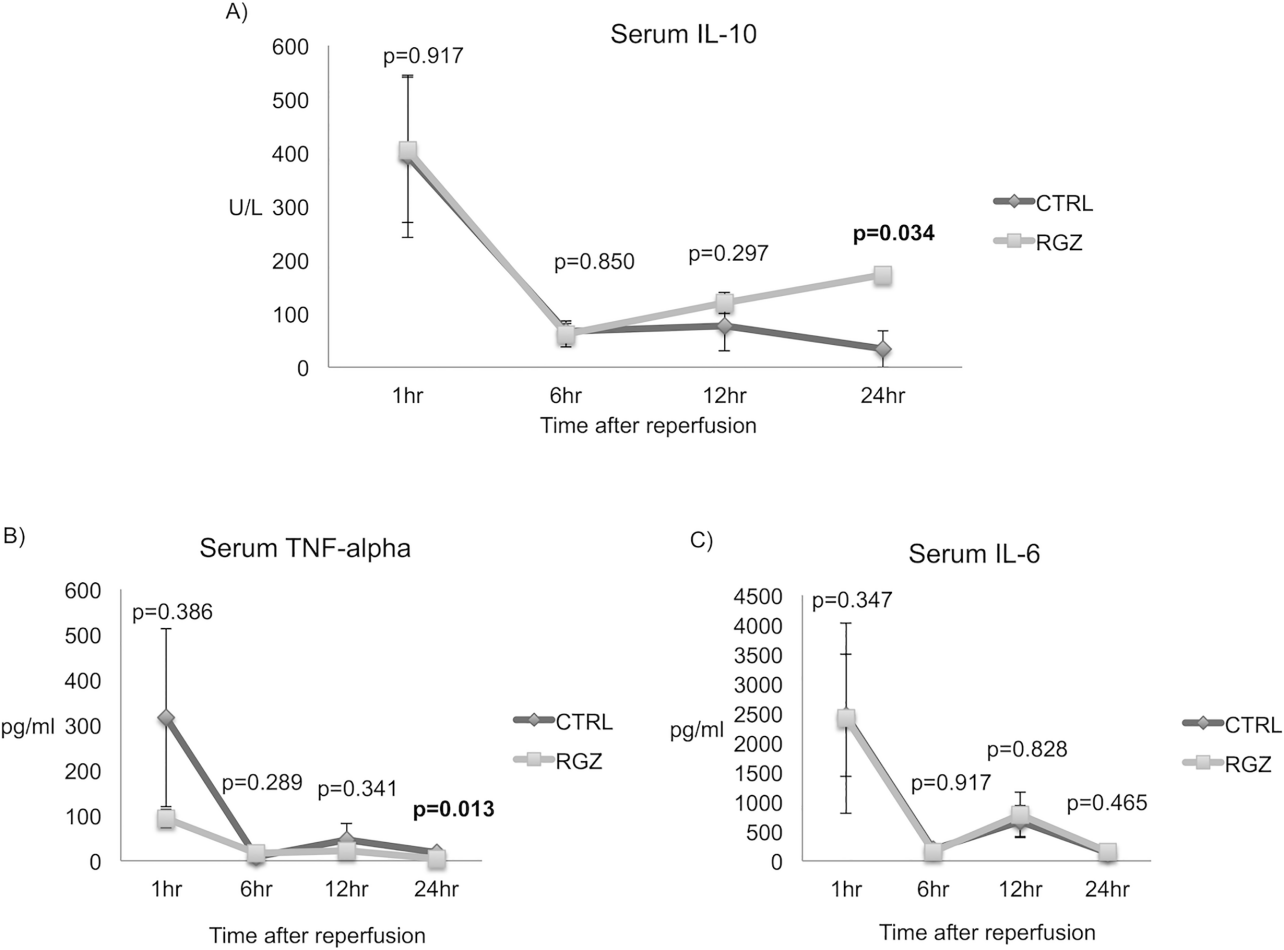
Serum cytokines. Anti-inflammatory cytokine IL-10 was found with higher levels in RGZ vs CTRL group 12hrs (119.98±46.22 vs 76.66±19.73 pg/ml; p = 297) following IRI difference became significant at 24hr time-point (172.64±34.18 vs 34.18±6.89 pg/ml; p = 0.034) (A). TNF-alpha showed lower levels since 1hr after reperfusion difference that became significant 24hr (4.15±0.95 vs 18.08±2.35pg/ml; p = 0.013) after IRI (B). Serum IL-6 levels were similar for both groups at all time-points (C). Abbreviations: TNF-alpha Tumor necrosis factor, IL Interleukin, CTRL Control, RGZ Rosiglitazone. Five experiments (n = 5) per group per time point were performed. Results are shown as mean ± SEM, Mann-Whitney-U test.

TNF-alpha was evaluated due to its roles as a critical regulator of the inflammatory response, a mediator of apoptotic pathways, and one of the cytokines released by pro-inflammatory KCs. Serum TNF-alpha levels were three times lower in the mice treated with RGZ versus control group at 1hr after reperfusion (93.04±20.08 vs 315.92±196.39 pg/ml; p = 0.386), this difference became significant 24hr after reperfusion (4.15±0.95 vs 18.08±2.35 pg/ml; p = 0.013). We also analyzed serum IL-10 for its anti-inflammatory effect and as an indirect marker of anti-inflammatory Kupffer cells. Serum IL-10 levels were comparable in the RGZ versus control group at 1hr and 6hr post-reperfusion (1hr: 405.10±151.44 vs 393.46±135.73 pg/ml; p = 0.917; 6hr: 61.56±14.73 vs 67.12±23.94 pg/ml; p = 0.850). Interestingly, we found that in a late phase post reperfusion IL-10 levels started increasing with higher values in the RGZ treated group when compared against control group by the hour 12 following IRI (119.98±46.22 vs 76.66±19.73 pg/ml; p = 0.297), this difference became significant 24hr after the ischemic event (172.64±34.18 versus 34.18±6.89 pg/ml; p = 0.034). Levels of IL-6 were comparable in both groups at the different time-points assessed (Fig 4)

### Effect of PPAR-γ activation on liver macrophages following IRI

**Fig 5:**
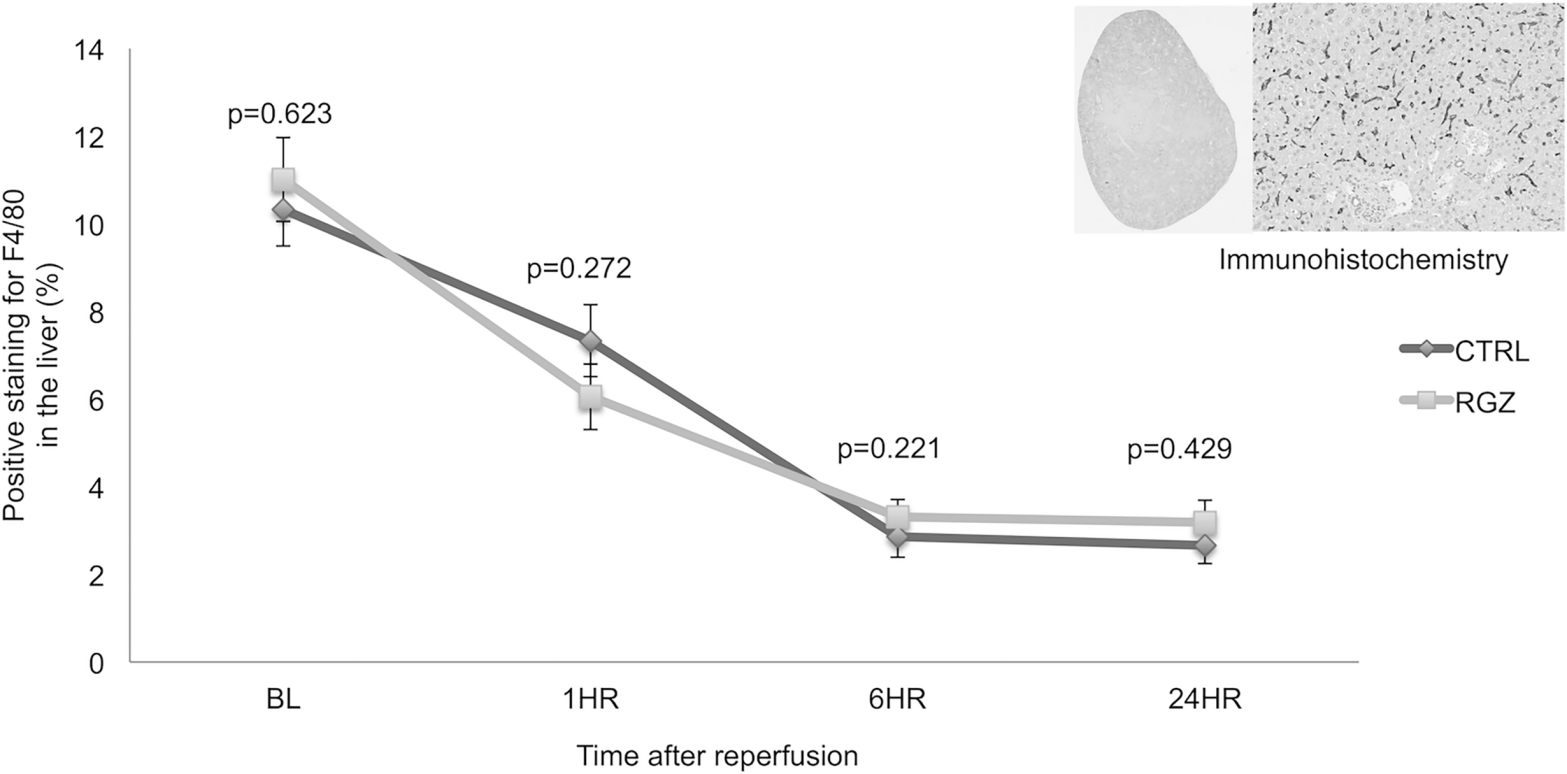
Hepatic macrophages. Immunohistochemistry technique showing how the total percentage of F4/80 staining decreases progressively after the IRI event. No difference was found when RGZ treated group was compared against the control group. Abbreviations: RGZ Rosiglitazone, CTRL Control. Five experiments (n = 5) per group per time point were performed. Results are shown as mean ± SEM, Mann-Whitney U test.

To explore the effect of RZG on liver macrophages, we investigated the total percentage of F4/80 staining prior and following the reperfusion event in both groups. Using image analysis, we found a progressive reduction of positive staining after reperfusion in both groups, with the lowest F4/80 expression 24hrs after the IRI event. Comparison between both groups showed no difference on the total percentage of positive staining in the RGZ treated group when compared with the control group at any time-point (BL: 11.01±0.96 versus 10.33±0.84%; p = 0.623; 1hr: 6.05±0.75% versus 7.33±0.81%; p = 0.272; 6hr: 3.31±0.40% versus 2.85±0.46%; p = 0.221; 24hr: 3.18±0.50% versus 2.65±0.41%; p = 0.429) (Fig 5). These results demonstrated that the IRI event by itself induces reduction of the total macrophages within the liver, and treatment with RGZ did not affect the percentage of positive staining.

### Pro-inflammatory nitric oxide+ Kupffer cells population is decreased by PPAR-γ activation

**Fig 6:**
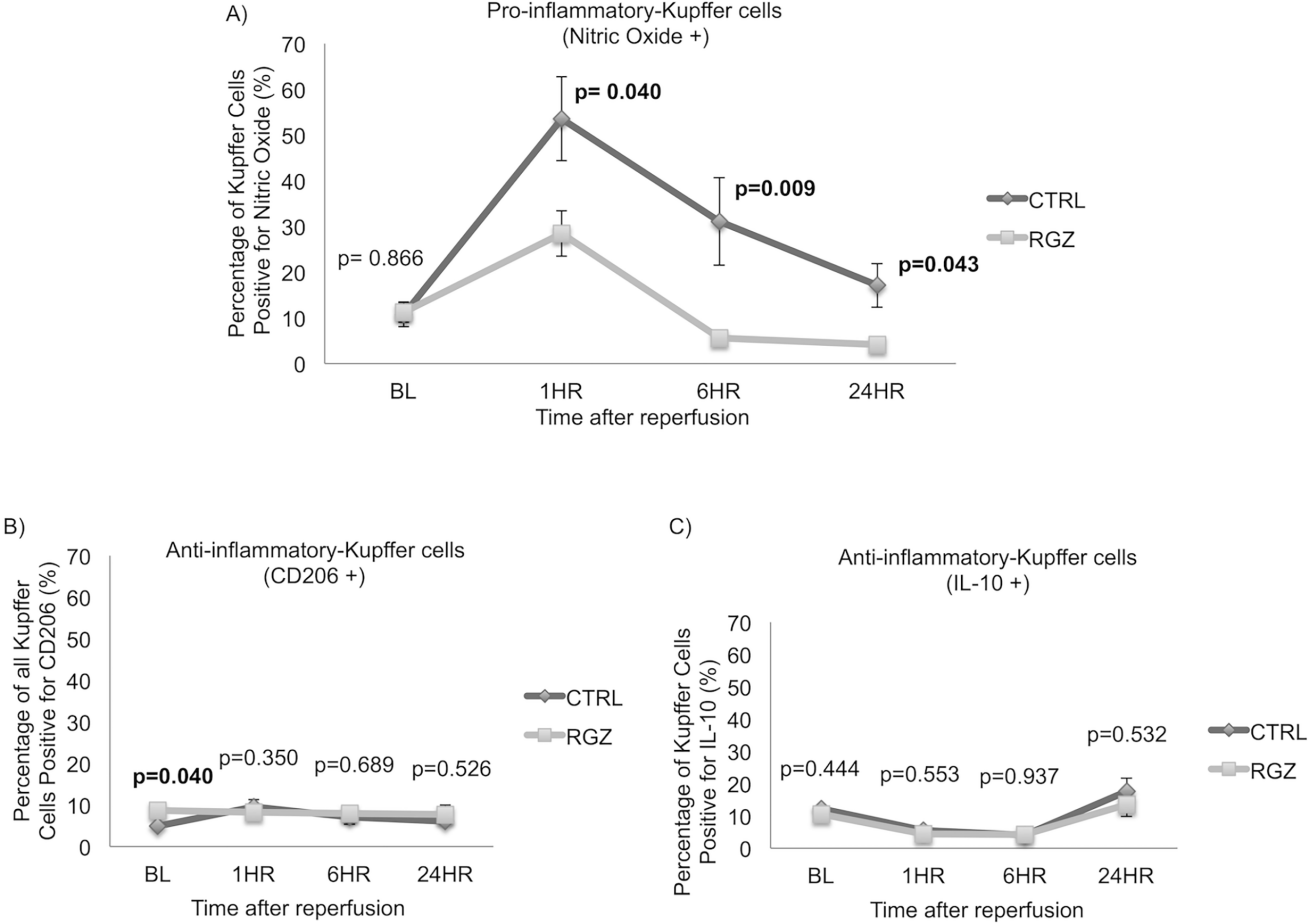
Kupffer cell populations. Flow-cytometry analysis showed a significant decrease on pro-inflammatory NO+ KCs population in RGZ vs CTRL group since 1hr (28.49±4.99 vs 53.54±9.15%; p = 0.040), this difference further increase at 6hr (5.51±0.54 vs 31.12±9.58%; p = 0.009) and 24hr (4.15±1.50 vs 17.10±4.77%; p = 0.043) following reperfusion (A). Analysis of anti-inflammatory CD206+ KCs population showed a significant increase in RGZ vs CTRL group prior to IRI (8.62±0.96 vs 4.88±0.50%; p = 0.040) (B). No difference in the percentage of anti-inflammatory IL10+ KCs was found prior or after reperfusion (C). Abbreviations: RGZ-Rosiglitazone, CTRL-Control. Five experiments (n = 5) per group per time point were performed. Results are shown as mean±SEM, Mann-Whitney-U-test.

We used flow-cytometry to explore the KC population and its pro-inflammatory NO+ phenotype activation after administration of PPAR- γ agonist. The percentage of pro-inflammatory NO+ KCs at baseline (prior to ischemia-reperfusion event) was comparable for RGZ versus the control group (11.27±2.24 vs 10.67±2.60%; p = 0.866). In contrast, pro-inflammatory NO+ KCs population was significantly reduced at 1hr post reperfusion in the RGZ treated group when compared with the control group (28.49±4.99 vs 53.54±9.15%; p = 0.040), this difference further increases at 6hr (5.51±0.54 vs 31.12±9.58%; p = 0.009) and 24hr (4.15±1.50 vs 17.10±4.77%; p = 0.043) following reperfusion (Fig 6).

### Anti-inflammatory CD206+ Kupffer cells population increases prior to IRI by the activation of PPAR-γ

We investigated anti-inflammatory CD206+ KCs population using flow-cytometry, prior to the ischemic insult the percentage of anti-inflammatory CD206+ KCs population was increased by 50% in the RGZ vs control group (8.62±0.96 vs 4.88±0.50%; p = 0.040). In contrast, the percentage of anti-inflammatory CD206+ KCs population ^+^ cells was comparable after reperfusion in the RGZ treated versus the control group (Fig 6). In addition to the surface marker (CD206), analysis for IL-10 ^+^ expression in Kupffer cells was performed to identify functional anti-inflammatory Kupffer cell polarization. Results showed that the percentage of anti-inflammatory Kupffer cells expressing IL-10 were comparable in both the RGZ treated versus control group at all the time-points prior and after IRI (BL: 10.60±1.88 vs 12.27±0.34%, p = 0.444; 1hr: 4.42±0.71 vs 5.44±1.36%; p = 0.553; 6hr: 4.23±1.47 vs 4.06±1.10%; p = 0.937, 24hr: 13.48±3.65 vs 17.65±4.05%; p = 0.532) (Fig 6).

### PPAR-γ activation reduces pro-inflammatory-NO+/ anti-inflammatory-CD206+ Kupffer cells ratio

**Fig 7:**
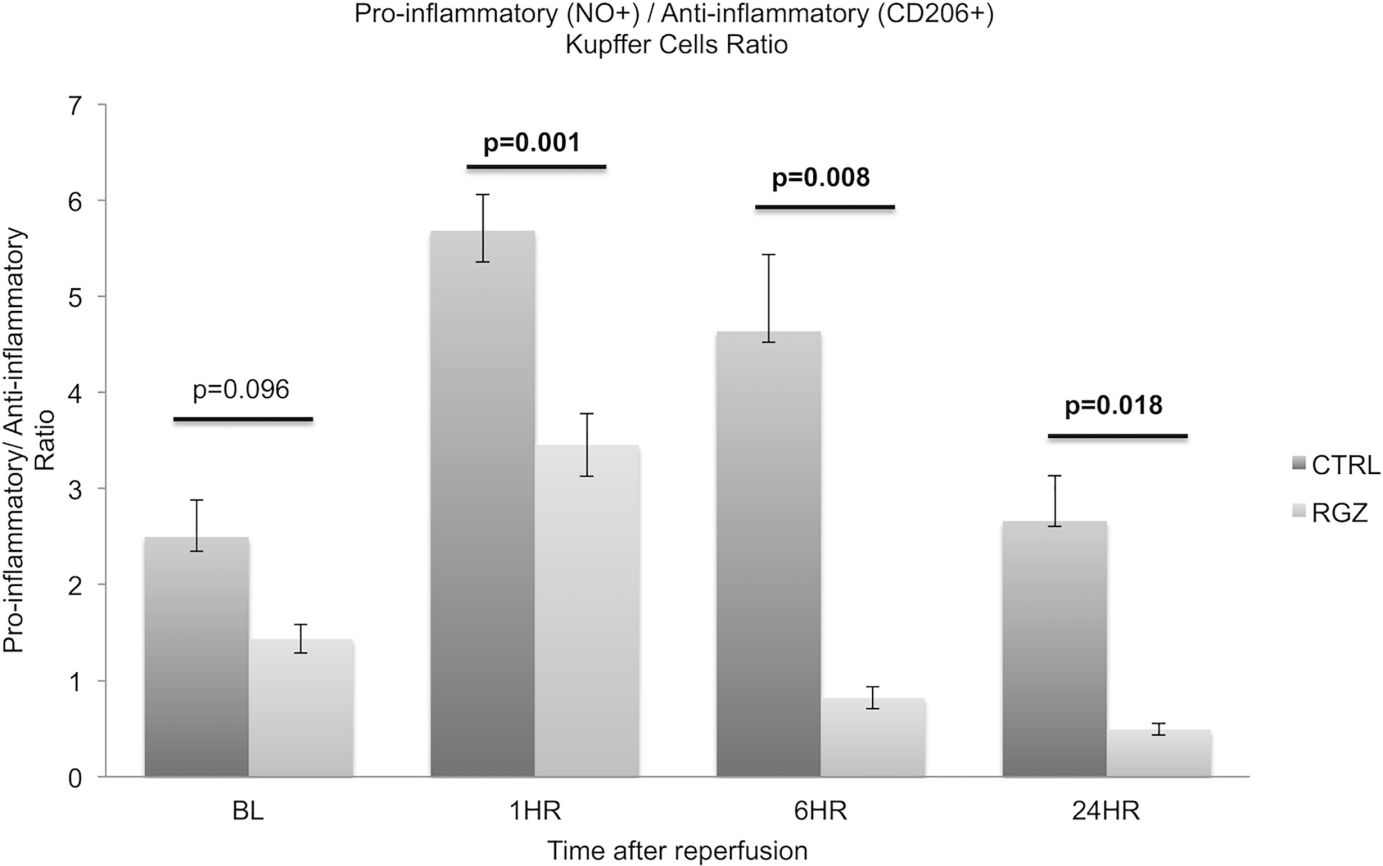
Pro-inflammatory-NO+/ anti-inflammatory-CD206+ Kupffer cells ratio prior and after reperfusion. Pro-inflammatory-NO+/ anti-inflammatory-CD206+ KCs ratio was lower in the RGZ treated group at all the studied time points. Significant lower ratio was found in the RGZ vs CTRL group since 1hr after reperfusion group (3.45±0.32 vs 5.68±0.37; p = 0.001), this difference remained significant at 6hr (0.81±0.11 vs 4.63±0.79; 0.008) and 24hr (0.49±0.05 vs 2.66±0.46; p = 0.018) post-reperfusion. Abbreviations: CTRL control, RGZ Rosiglitazone. Five experiments (n = 5) per group per time point were performed. Results are shown as mean ± SEM, Mann-Whitney U test.

To explore the predominant Kupffer cell activity at every time point we determined the pro-inflammatory-NO+/ anti-inflammatory-CD206+ KCs ratio. While no difference was observed between the RGZ vs control group at baseline, (1.43±0.14 vs 2.49±0.38; p = 0.096) a significantly lower ratio was seen as early as 1hr post reperfusion in the RGZ versus the control group (3.45±0.32 vs 5.68±0.37; p = 0.001). These differences remained significant at 6hr (0.81±0.11 vs 4.63±0.79; 0.008) and 24hr (0.49±0.05 vs 2.66±0.46; p = 0.018) post-reperfusion (Fig 7).

### PPAR-γ antagonist reverses beneficial effect on liver injury, hepatocellular apoptosis, and pro-inflammatory NO+ Kupffer cells population

**Fig 8:**
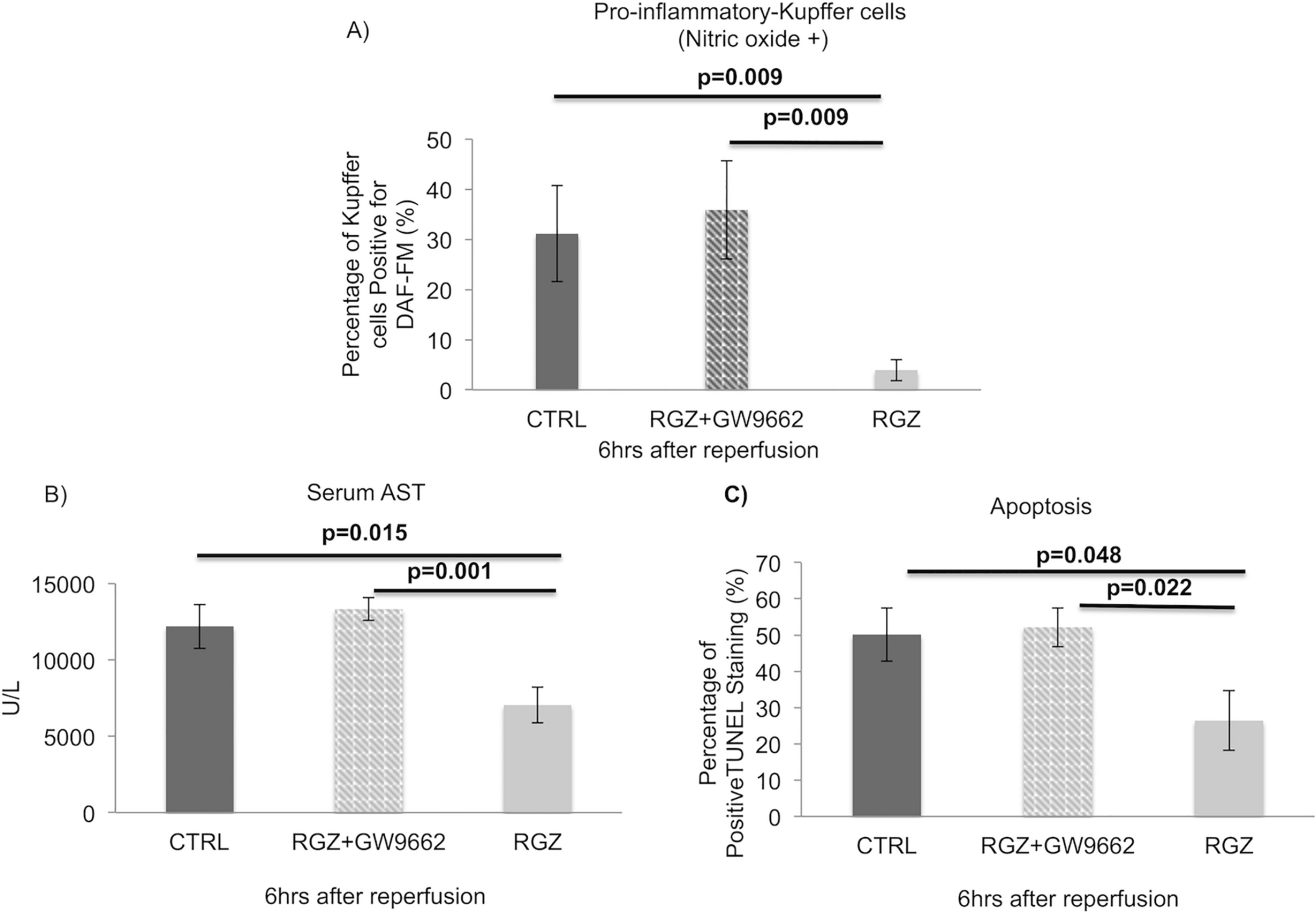
PPAR-γ antagonist-6hrs after reperfusion. Flow-cytometry shows how the effect on pro-inflammatory NO+ KC polarization was significantly blocked by the use of PPAR-γ antagonist (GW9662) (RGZ: 3.93±3.61%, RGZ+GW9662: 35.86±21.85%, Control: 31.12±21.42%, p = 0.009) (A). Serum AST levels were also significantly reversed when antagonist was included as an intervention (B). The grade of apoptosis was significantly increased with the use of antagonist in combination with RGZ when compared with RGZ alone (C). Abbreviations: DAF-FM 4-amino-5-methylamino-2’,7’-Difluoroflurescein, TUNEL-Terminal deoxynucleotidyl transferase (TdT) dUTP Nick-End Labeling, RGZ-Rosiglitazone, CTRL-Control. Five experiments (n = 5) per group per time point were performed. Results are shown as mean ± SEM, Mann-Whitney-U-test.

As an additional approach, was utilized a PPAR-γ receptor antagonist agent, GW9662 to investigate if the blockage of PPAR-γ receptor could reverse the favorable effect found with PPAR-γ activation and to exclude possible cross-activation on PPAR-alpha and delta receptors. For this set of experiments, we chose the 6hr post reperfusion time point when the strongest protective effect of RZG was observed. We examined Kupffer cell population and found that the treatment with GW9662 prior to the administration of RZG increased the percentage of pro-inflammatory NO+ KCs to levels that were higher than the observed in the control group (RGZ: 3.93±3.61%, RGZ+GW9662: 35.86±21.85%, Control: 31.12±21.42%, p = 0.009). Moreover, the favorable effect of RZG previously observed on hepatic injury and apoptosis were also reversed when GW9662 was administered (Fig 8).

### PPAR-γ agonist treatment following reperfusion does not decrease hepatic injury and apoptosis

**Fig 9:**
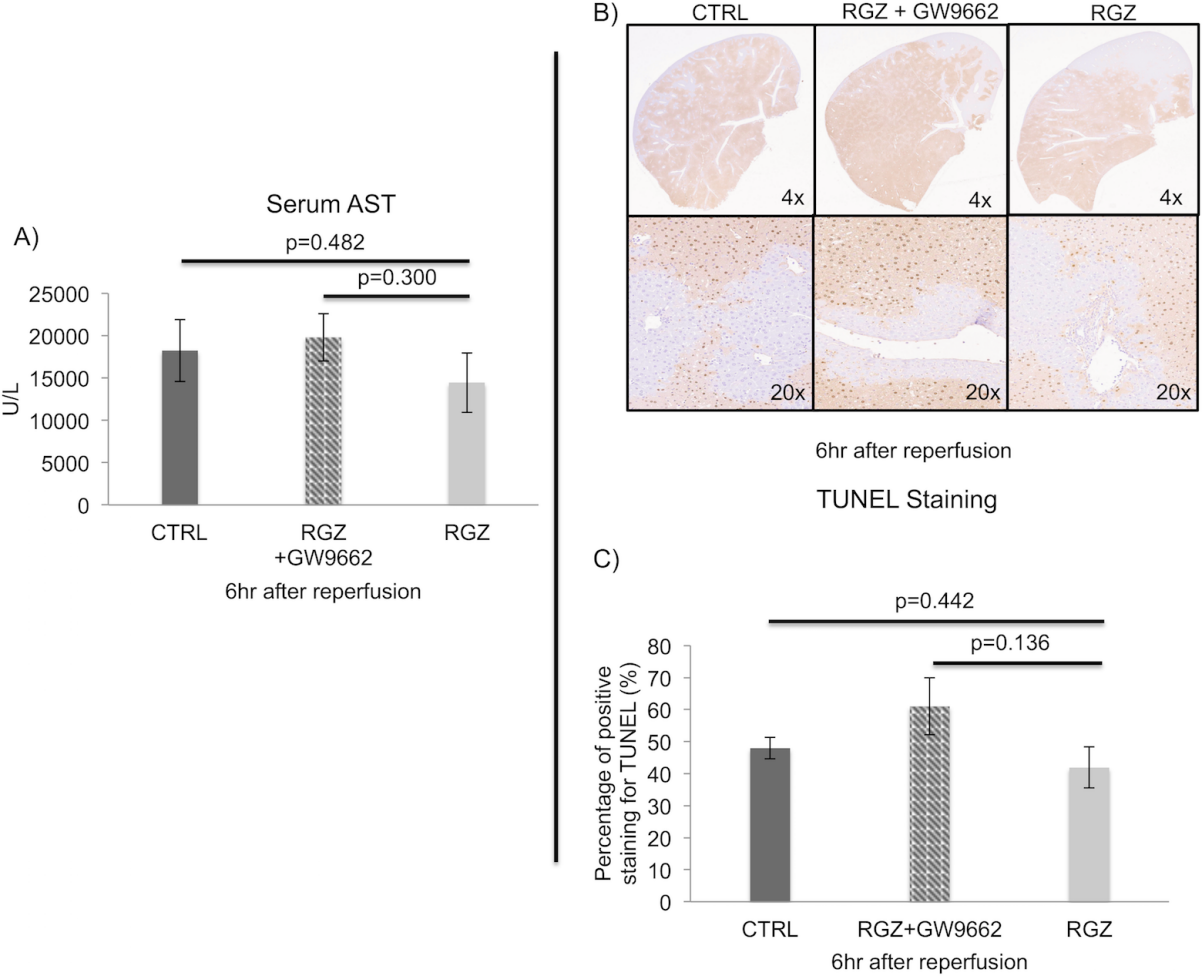
Hepatic injury and apoptosis in RGZ treated mice following reperfusion. Analysis of hepatic injury showed no significant difference in the RGZ vs Control group for AST (14451±3518 vs 18231±3688U/L, p = 0.482), Inhibition of PPAR-γ with GW9662 increased further the levels of AST when compared with control group (A). Representative images of TUNEL immunohistochemistry showing a similar grade of apoptosis in RGZ, CTRL and RGZ+GW-9662 groups at 6hr post-reperfusion (B). TUNEL positive staining was found similar in RGZ vs control group at 6hr post-reperfusion (42±6 vs 48±3%, p = 0.442) (C). GW-9662 showed a reversal of the slight beneficial effect on apoptosis found in the RGZ group (C). Note: Staining was assessed with image software analysis. Abbreviations: AST-aspartate aminotransferase, RGZ-Rosiglitazone, CTRL-Control, TUNEL-Terminal deoxynucleotidyl transferase(TdT) dUTP Nick-End-Labeling. Five experiments (n = 5) per group per time point were performed. Results are shown as mean ± SEM, Mann-Whitney U test.

An additional set of experiments was performed to evaluate the effectiveness of treatment with RGZ following reperfusion. For these experiments, the samples were collected 6hrs after reperfusion. The administration of RGZ as a single dose showed no significant decrease in the RGZ vs control group for serum AST levels (14451±3518 vs 18231±3688U/L, p = 0.482) after 6 hours of reperfusion. Further analysis was performed to evaluate apoptosis and results from the software image assessment demonstrated a no significant decrease the RGZ vs Control group for TUNEL staining (42±6 vs 48±3%, p = 0.442). The use of GW9662 prior to the injection of RGZ reversed the slight beneficial effect on hepatic injury and apoptosis level secondary to treatment with RGZ (Fig 9).

## Discussion

In this study, we assessed the effects of PPAR-γ agonist administration prior to hepatic ischemia-reperfusion injury in a mouse model. Our findings demonstrated that the PPAR-γ activation prior to IRI decreases hepatic injury, as determined by hepatic enzymes and markers of apoptosis and necrosis. Furthermore, PPAR-γ agonist induced the up-regulation of IL-10 and down-regulation of TNF-alpha accompanied with a reduction of NO positive-Kupffer cells after reperfusion. Our findings indicate that apart from the decrease on ischemia-reperfusion injury the PPAR-γ activation reduces the pro-inflammatory NO positive Kupffer cell phenotype.

Preceding studies in cerebral, cardiac, renal and liver tissue had demonstrated that the use of PPAR-γ agonist could ameliorate IRI. The potential mechanisms that had been described from these studies were the decrease of expression of matrix metalloproteinases, increase on superoxide dismutase 1, upregulation of AMP-activated protein kinase and Akt, but also the downregulation of JNK pathway signaling [43],[44]. Additionally, these studies have reported that PPAR-γ activation decreases the expression of TNF-alpha, IL-1Beta and the migration of neutrophils. The effects of PPAR-γ activation on hepatocytes have previously been investigated in in vivo and in vitro inflammation models. Results from these studies showed a reduction of Cxcl1 and IL-8 expression (key chemokines for neutrophil recruitment) in hepatocytes treated with PPAR-γ agonist prior to inflammation. Furthermore, these studies demonstrated that PPAR-γ agonist treatment was able to attenuate the suppression of bile acid transporters and Cyp3a11 expression secondary to inflammation. In addition, PPAR-γ activation lead to the maintenance of nuclear RXR alpha levels [45], [46] which has been linked to liver regeneration [47]. PPAR-γ agonist has also demonstrated a positive effect on endothelial cell proliferation after PPAR-γ activation in livers treated prior to inflammation [48]. Of interest, no prior study had investigated the role of PPAR-γ activation on Kupffer cell polarization in liver ischemia-reperfusion injury.

To our knowledge, this is the first study in a model of hepatic IRI where the Kupffer cell polarization has been evaluated following PPAR-γ activation. We determined Kupffer cell polarization by flow-cytometry identifying the polarization of this population after hepatic reperfusion and the effects that PPAR-γ activation has on pro-inflammatory, anti-inflammatory Kupffer cell phenotypes.

In our study, even though we found a significant difference on the anti-inflammatory CD206+ Kupffer cell population prior to the event of IRI this difference was not kept after reperfusion. In contrast, the pro-inflammatory NO+ Kupffer cell population was significantly and consistently diminished after reperfusion. Our findings contrast with what has been found in models of IRI for other tissues (brain and kidney) where the main effect of the PPAR-γ activation was the increase on anti-inflammatory macrophage population [49],[50],[51]. Moreover, according to our results on pro-inflammatory-NO+/ anti-inflammatory-CD206+ Kupffer cells ratio the high predominance of pro-inflammatory NO+ activity is associated with an increased IRI and the reversal of this ratio by PPAR-γ activation as early as 6hrs after reperfusion associated with an improvement on the outcomes of inflammatory response, hepatic injury, and necrosis.

We also investigated the effects of a PPAR-γ antagonist in combination with PPAR‒γ administration on the peak injury at 6hrs after reperfusion. Interestingly, we found that PPAR-γ receptor antagonist administration resulted in an increased hepatocyte injury, apoptosis, and the pro-inflammatory NO+ Kupffer cell population when compared to the untreated control group. It is possible that the increased injury with PPAR-γ receptor antagonist administration results from the additional blockage of endogenous PPAR-γ activation [52]. This suggests that the PPAR‒γ pathway could have an important internal protective auto-regulatory mechanism to diminish the exaggerated inflammatory response following IRI. In addition, the reversal of the protective effects from PPAR-γ by its receptor antagonist demonstrates that the positive effect found by the use of PPAR-γ is primarily due to the activation of this specific receptor and not alternative activation of other peroxisome proliferator-activated receptors

Our findings could have important clinical implications. In the setting of liver transplantation, the time point of IRI is known in advance. PPAR-γ activation could be achieved prior to the ischemic insult by treating the donor before organ retrieval. In addition, normothermic ex vivo liver perfusion has recently been established as a novel organ preservation technique. Utilizing a PPAR-γ agonist during normothermic ex vivo liver perfusion could induce resistance of the graft against reperfusion injury at the time of transplantation.

Our study has several limitations. First, effects of PPAR-γ receptor activation on intracellular mediators of inflammation were not investigated. Second, effects of cold vs warm ischemic injury on PPAR-γ mediated reperfusion injury remain unclear. Furthermore, in our study the PPAR-γ agonist was only administered prior to the ischemic insult. Repeat administration of PPAR-γ could have additional benefits on regeneration and organ repair. It is important to highlight that the results of this study cannot directly link the changes in macrophage polarization to the improved outcomes after PPAR-γ agonist treatment. But our findings provide a novel rationale for future studies to directly examine the causal relationship.

In summary, this study emphasizes the importance of Kupffer cells polarization during the initial response to hepatic IRI. Modification of Kupffer cell polarization could be a novel strategy to reduce the propagation of reperfusion injury and improve liver function following transplantation and surgery.

## Conclusion

PPAR-γ reduces hepatic IRI and decreases the pro-inflammatory population of Kupffer cells. PPAR-γ activation prior to reperfusion can become an important tool to decrease the pro-inflammatory Kupffer cell population and improve the outcomes of liver ischemia and reperfusion injury in liver surgery.

## Abbreviations

AST: Aspartate aminotransferase
BL: Baseline
CTRL: Control
NO: Nitric Oxide
U/L: units per liter
IRI: Ischemia-reperfusion injury
KC: Kupffer cell
POD: Post-operative day
RGZ: Rosiglitazone
TUNEL: Terminal deoxynucleotidyl transferase dUTP nick end labeling
